# Structural Analysis of Gluco-Oligosaccharides Produced by *Leuconostoc lactis* and Their Prebiotic Effect

**DOI:** 10.3390/molecules24213998

**Published:** 2019-11-05

**Authors:** Sulhee Lee, Jisun Park, Jae-Kweon Jang, Byung-Hoo Lee, Young-Seo Park

**Affiliations:** 1 Department of Food Science and Biotechnology, Gachon University, Seongnam 13120, Korea; sulhee2340@gmail.com (S.L.); jisunny4424@gmail.com (J.P.); blee@gachon.ac.kr (B.-H.L.); 2 Research Group of Healthcare, Korea Food Research Institute, Wanju 55365, Korea; 3 Food Nutrition Major, School of Food, Chungkang College of Cultural Industries, Icheon 17390, Korea; jkjang@chungkang.ac.kr

**Keywords:** prebiotics, probiotics, oligosaccharides, lactic acid bacteria, NMR

## Abstract

*Leuconostoc lactis* CCK940, which exhibits glycosyltransferase activity, produces oligosaccharides using sucrose and maltose as donor and receptor molecules, respectively. The oligosaccharides produced were purified by Bio-gel P2 chromatography and the purified oligosaccharides (CCK-oligosaccharides) consisted of only glucose. ^1^H-NMR analysis revealed that the CCK-oligosaccharides were composed of 77.6% α-1,6 and 22.4% α-1,4 glycosidic linkages, and the molecular weight of the CCK-oligosaccharides was found to be 9.42 × 10^2^ Da. To determine the prebiotic effect of the CCK-oligosaccharides, various carbon sources were added in modified media. Growth of six probiotic strains, *Lactobacillus casei*, *L. pentosus*, *L. plantarum*, *Weissella cibaria*, *Bifidobacterim animalis*, and *Saccharomyces cerevisiae*, was better when the CCK-oligosaccharides were used as the sole carbon source compared to fructo-oligosaccharides, which are widely used as prebiotics. These results showed that the CCK-oligosaccharides produced from *Leu. lactis* CCK940 could serve as good candidates for novel prebiotics.

## 1. Introduction 

Lactic acid bacteria produce carbohydrates, glycosides, oligosaccharides, or polysaccharides through glycosyltransferase activity [1,2]. Glycosyltransferase effectively synthesizes oligosaccharides such as galacto-oligosaccharides (GOS) and fructo-oligsoaccharides (FOS), which are widely used as prebiotics in industry [3,4,5]. The types of product synthesized by glycosyltransferase can be classified into dextransucrase (EC 2.4.1.5), alternansucrase (EC 2.4.1.140), mutansucrase (EC 2.4.1.5), and reuteransucrase (EC 2.4.1.5) [6,7]. The various oligosaccharides, which are synthesized by these enzymes, have linear chains which are composed of d-glucopyranosyl units. The dextran polymer has α-1,6 glycosidic bonds, mutan polymer has α-1,3 glycosidic bonds, alternan polymer consists of alternate α-1,6 and α-1,3 glycosidic bonds, and reuteran polymer has α-1,4 glycosidic bonds [7,8]. 

Prebiotics are typically defined as “selectively fermented, non-digestible food ingredients or substances that specifically support the growth and/or activity of health-promoting bacteria that colonize the gastrointestinal tract” [9]. This definition of prebiotics demonstrates that not all dietary carbohydrates are prebiotics, but they must satisfy the following criteria: (1) resistance to gastric acidity (low pH), hydrolysis by mammalian enzymes, and gastrointestinal absorption; (2) fermentation by intestinal microflora; and (3) selective stimulation of the growth and/or activity of the intestinal bacteria that contribute to health and well-being [10,11]. Prebiotics typically contain FOS, GOS, isomalto-oligosaccharide (IMO), and cyclo-oligosaccharides. Many studies have shown that oligosaccharides as prebiotics exert positive effects on the human body. They improve intestinal function, stimulate the growth of beneficial bacteria, inhibit the growth of pathogens, increase short chain fatty acids (SCFAs), reduce total cholesterol and low-density lipoprotein (LDL) cholesterol, and enhance immune function [12,13,14,15]. 

In our previous reports, *Leuconostoc lactis* CCK940, which has high glucansucrase activity, was isolated from home-made kimchi (Korean traditional fermented vegetables) and the production of oligosaccharides from *Leu. lactis* CCK940 was optimized [14,16]. The purified oligosaccharides from *Leu. lactis* CCK940 showed immunostimulating effects on RAW264.7 macrophage cells [14].

Because oligosaccharides are well known prebiotics, it was predicted that the oligosaccharides produced by *Leu. lactis* CCK940 would also have prebiotic effects. Since only limited studies examining the prebiotic effects of oligosaccharides produced by lactic acid bacteria have been conducted, in the present study, we investigated the prebiotic effects of the CCK-oligosaccharides produced by *Leu. lactis* CCK940 using 24 probiotic strains.

## 2. Results 

### 2.1. Analysis of the Carbohydrate Composition of the CCK-Oligosaccharides

**Figure 1:**
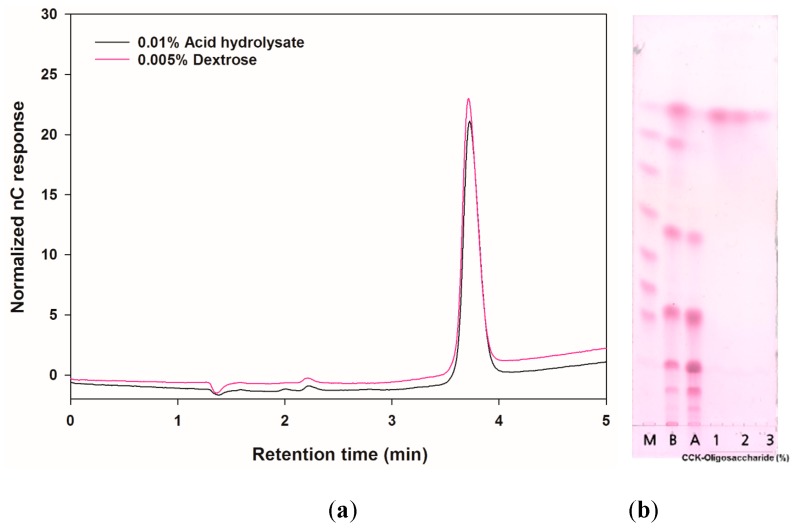
High-performance anion exchange chromatography with pulsed amperometric detection (HPAEC-PAD) and thin-layer chromatography (TLC) chromatograms of CCK-oligosaccharide (produced by *L. lactis* CCK940) acid hydrolysate. (**a**) HPAEC-PAD chromatogram of 0.005% (*w*/*v*) dextrose and 0.01% acid hydrolysate. (**b**) TLC chromatograms obtained before and after purification of CCK-oligosaccharides and acid-hydrolyzed CCK-oligosaccharides (M: glucose polymers G1–G7; B: before purification; A: after purification by Bio-gel P2; 1: 1% of acid hydrolysate; 2: 0.5% of acid hydrolysate; 3: 0.2% of acid hydrolysate).

To analyze the sugar composition, the CCK-oligosaccharides were acid-hydrolyzed by trifluoroacetic acid (TFA) and their hydrolyzed products were identified using thin-layer chromatography (TLC) and high-performance anion exchange chromatography with pulsed amperometric detection (HPAEC-PAD). As shown in Figure 1, the CCK-oligosaccharides were completely hydrolyzed by TFA; the HPAEC-PAD chromatograms of 0.005% (*w*/*v*) dextrose and hydrolysates were identical. The purified oligosaccharides were identified as gluco-oligosaccharides (CCK-oligosaccharides) consisting of only glucose. 

**Figure 2:**
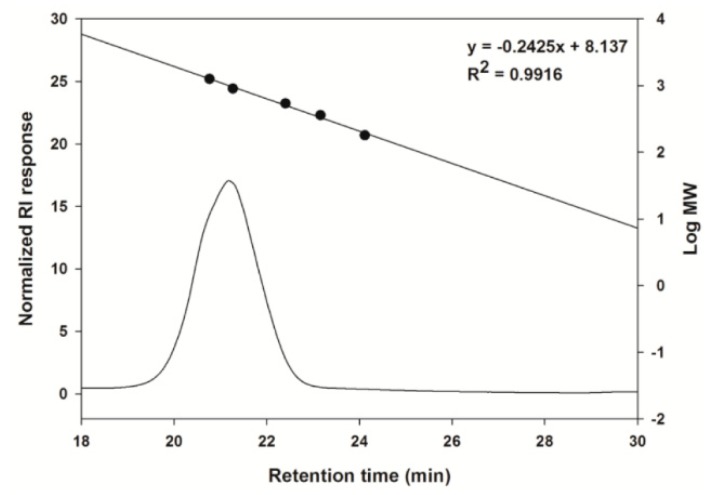
Size exclusion high-performance liquid chromatography (HPLC) chromatogram of the major CCK-oligosaccharides.

The molecular weight of the purified CCK-oligosaccharides was analyzed using size exclusion chromatography (Figure 2). Among the oligosaccharides that comprised the CCK-oligosaccharides, the molecular weight of the highest concentration oligosaccharide was determined to be 9.42 × 10^2^ Da. 

### 2.2. Determination of the Types of Glycosidic Bonds

**Figure 3:**
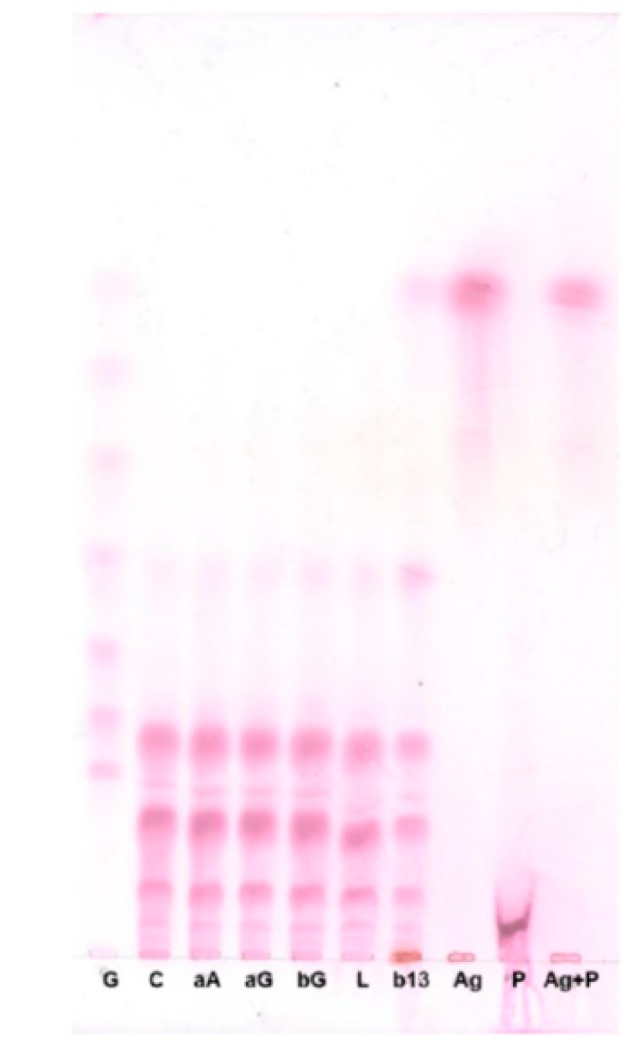
TLC chromatogram of enzymatic analysis of CCK-oligosaccharides. G: G1–G7 standard; C: CCK-oligosaccharides; aA: CCK-oligosaccharides treated with α-amylase; aG: CCK-oligosaccharides treated with α-glucosidase; bG: CCK-oligosaccharides with β-glucosidase; L: CCK-oligosaccharides treated with lichenase; b13: CCK-oligosaccharides treated with β-1,3-d-glucanase; Ag: CCK-oligosaccharides treated with amyloglucosidase; P: CCK-oligosaccharides treated with pullulanase M1; Ag+P: CCK-oligosaccharides treated with amyloglucosidase and pullulanase M1.

To determine the types of glycosidic bonds in the CCK-oligosaccharides, the CCK-oligosaccharides were hydrolyzed with several carbohydrate hydrolysis enzymes and their products were analyzed by TLC (Figure 3). CCK-oligosaccharides were not hydrolyzed by α-amylase, α-glucosidase, β-glucosidase, lichenase, or β-1,3-d-glucanase. CCK-oligosaccharides were hydrolyzed by pullulanase M1 and produced oligosaccharides with a degree of polymerization (DP) of 11. 

### 2.3. Analysis of the Linkage Ratio by ^1^H-NMR Spectroscopy

**Figure 4:**
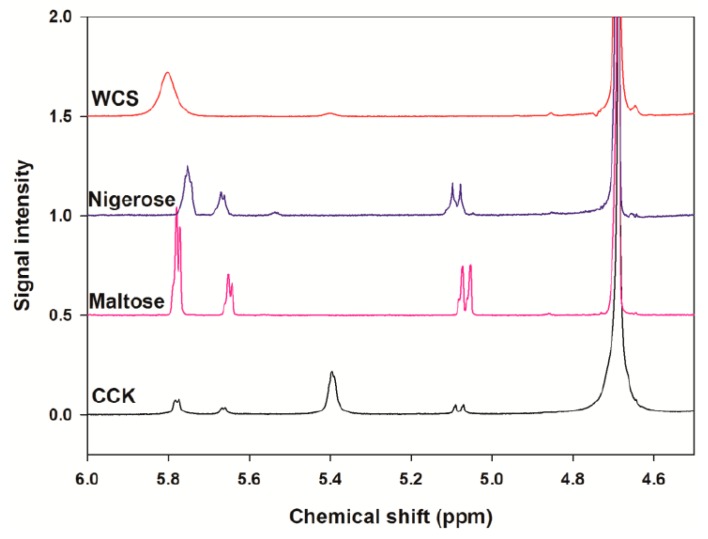
Comparison of the ^1^H-NMR spectra (400 MHz, D_2_O) for CCK-oligosaccharides, maltose, nigerose, and waxy corn starch (WCS) (from the bottom).

^1^H-NMR spectroscopy was used to analyze the linkage ratios of the CCK-oligosaccharides. Chemical shifts of CCK-oligosaccharides, maltose, nigerose, and waxy corn starch (WCS) were determined at 5–5.8 ppm (Figure 4). It was found that CCK-oligosaccharides were composed of only α-linkages. WCS was used as a standard for determining the chemical shift of the resonances from the ^1^H positions of α-1,4 and α-1,6 linkages. The results indicated that the percentages of the linkages for the CCK-oligosaccharides were 22.4% α-1,6 linkages and 77.6% α-1,6 linkages, and the branching ratio of α-1,4 to α-1,6 was calculated to be 0.3 (Table 1). 

### 2.4. Prebiotic Effects of the CCK-Oligosaccharides

**Figure 5:**
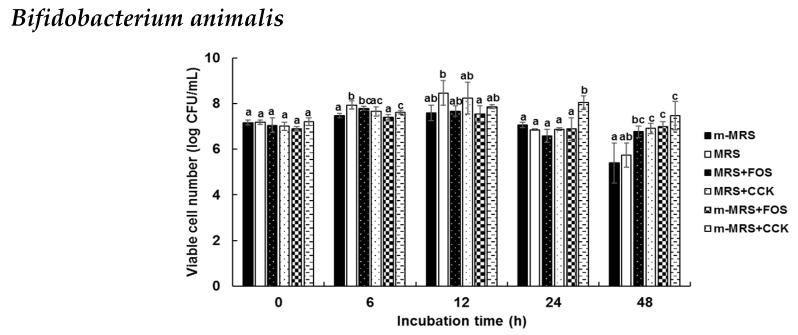

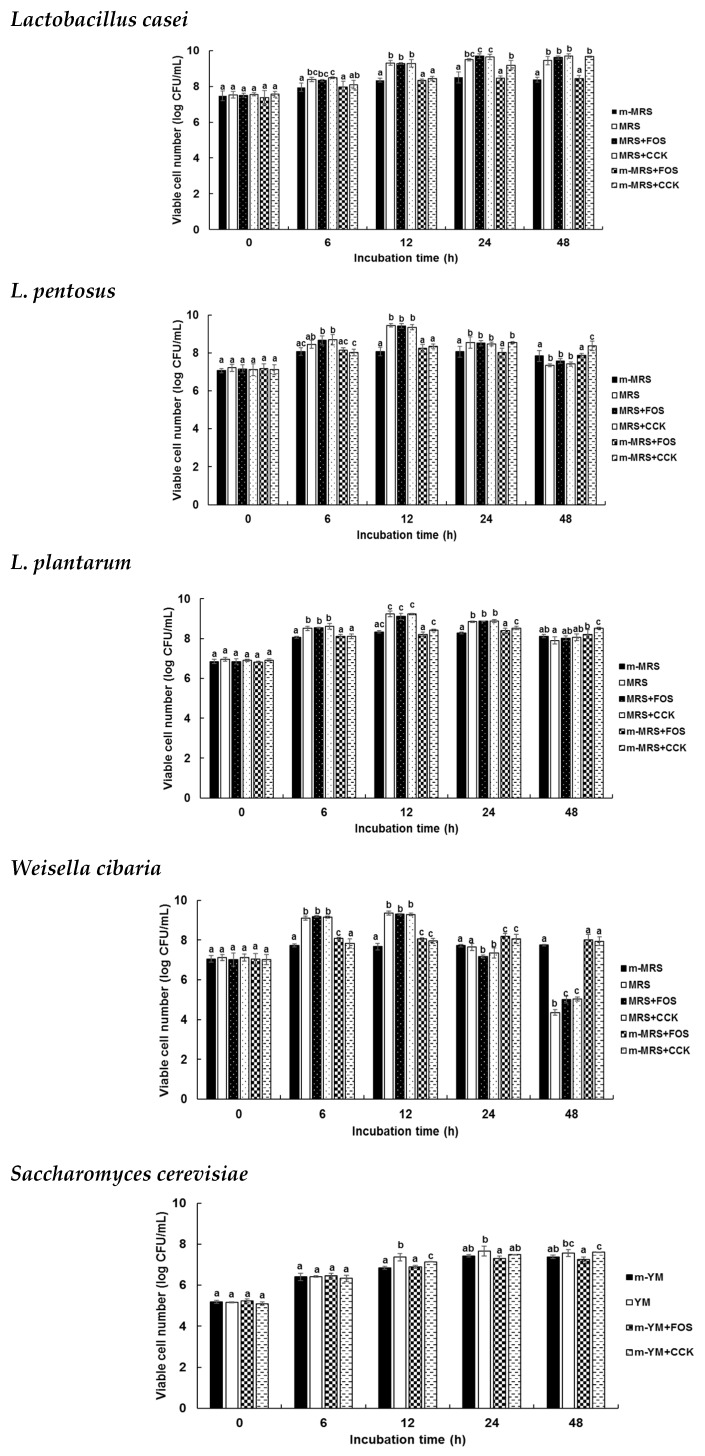
Prebiotic effects of CCK-oligosaccharides. One-way ANOVA was used for comparison of group mean values, followed by Duncan’s multiple range test for significance of individual comparisons (*p* < 0.05). Different alphabet letters among groups represent statistically significant difference.

To determine the prebiotic effects of the CCK-oligosaccharides, various carbon sources were added into modified media. Six of 24 strains, *Lactobacillus casei*, *L. pentosus*, *L. plantarum*, *Weissella cibaria*, *Bifidobacterim animalis*, and *Saccharomyces cerevisiae* were used to investigate CCK-oligosaccharides as prebiotics, compared to FOS which was used as a positive control (Figure 5). Viable cell numbers of *L. casei*, *L. pentosus*, and *L. plantarum* significantly increased in CCK media when compared with modified deMan, Rogosa, and Sharpe (MRS) (m-MRS) with FOS at 24 and 48 h of incubation. Viable cell numbers of *W. cibaria* were 9.36 and 4.35 log CFU/mL in MRS at 12 and 48 h, respectively. Viable cell numbers of *W. cibaria* were 8.01 and 7.94 log CFU/mL in FOS and CCK media, respectively, at 48 h of incubation. *B. animalis* also exhibited results similar to *W. cibaria*; the viable cell number of *B. animalis* in the CCK medium was significantly higher than that in FOS media. *B. animalis* and *S. cerevisiae* grew better in media containing CCK-oligosaccharides than in FOS. 

## 3. Discussion

It has been reported that *Leu. mesenteroides* B-742 produces GOS and mannitol using maltose as an acceptor [17], and that *Leu. mesenteroides* NRRL B-23188 synthesizes gluco-oligosaccharides using calcium hydroxide–sucrose solution [3]. Although many studies on the synthesis of gluco-oligosaccharides by *Leu. mesenteroides* have been conducted, no studies have been conducted to investigate the gluco-oligosaccharides synthesized by *Leu. lactis*.

It has been reported that when pooled human milk oligosaccharides are fractionated, the molecular weight of the fractions is 2.094–3.626 × 10^3^ Da [18], and dextransucrase catalyzes the synthesis of oligosaccharides in the range of 50–100 × 10^3^ Da [19]. The molecular weight of CCK-oligosaccharides was lower than that of other oligosaccharides.

α-Amylase (EC 3.2.1.1) hydrolyzes the α-1,4 glucose linkages of starch through an endo-type mechanism and α-glucosidase (EC 3.2.1.20) cleaves a glucose unit from the non-reducing end of α-1,4 oligosaccharides and polysaccharide starch fragments through an exo-type mechanism. CCK-oligosaccharides were not hydrolyzed by α-amylase or α-glucosidase, which implies that these carbohydrates are non-digestible in the human intestinal tract because α-amylase and α-glucosidase are major carbohydrate-hydrolyzing enzymes in the human digestive system. β-Glucosidase (EC 3.2.1.21) releases a glucose unit from the non-reducing end of β-1,4 or β-1,3 glycosidic bonds of oligosaccharides or polysaccharide fragments, lichenase hydrolyzes β-1,4 glycosidic linkages in β-glucans containing β-1,3 and β-1,4 bonds, and β-1,3-d-glucanase (EC 3.2.1.6) cleaves β-1,3 linkages. CCK-oligosaccharides were not hydrolyzed by these three enzymes. Amyloglucosidase (EC 3.2.1.3) hydrolyzes α-1,4 and α-1,6 glycosidic bonds from the non-reducing end of starch oligomers and polymers through an exo-type mechanism, and this enzyme hydrolyzed CCK-oligosaccharides and released glucose. Pullulanase M1 is an endo-type enzyme similar to amyloglucosidase, which hydrolyzes α-1,6 bonds of pullulan, amylopectin, glycogen, and α- and β-limit dextrins of amylopectin and glycogen. CCK-oligosaccharides were hydrolyzed by pullulanase M1 and produced oligosaccharides with a degree of polymerization (DP) of 11. Based on these results, it was predicted that there are no β-1,3 or β-1,4 glycosidic linkages in the CCK-oligosaccharides.

Nuclear magnetic resonance (NMR) is the most powerful tool available for evaluating oligosaccharide linkage ratios [20]. Generally, the chemical shift of an anomeric proton was detected at 4.3–5.9 ppm in the ^1^H-NMR spectra. Maltose and nigerose are disaccharides formed from two units of glucose joined with an α-1,4 bond and connected with an α-1,3 linkage, respectively. Waxy corn starch (WCS) consists of polysaccharides formed from glucose polymers joined with α-1,4 and α-1,6 bonds. The enzyme 4,6-α-glucanotransferase of *L. reuteri* 121 acts on malto-oligosaccharides (MOS); the MOS consist of α-1,4 and α-1,6 glycosidic linkages [21]. Dextran and alternan were produced by *Leu. mesenteroides* NRRL B-512F; dextran contains approximately 95% α-1,6 linkages; however, alternan contains alternating α-1,3 and α-1,6 linkages [22]. The structure of CCK-oligosaccharides was different from other oligosaccharide in that branching ratio of α-1,4 to α-1,6 was lower than others.

When the prebiotic effect of a sugar is examined, minimal medium without a carbon source can be used so that the supplemented sugar is the sole carbon source. However, because lactic acid bacteria are fastidious bacteria, there is no suitable minimal medium for the growth of lactic acid bacteria. MRS medium, which is usually used for the cultivation of lactic acid bacteria, is a complex medium, but the beef extract and yeast extract in MRS are utilized as nitrogen sources rather than carbon sources by lactic acid bacteria [23]. Therefore, use of MRS medium without glucose is alternative for the examination of prebiotic effects. Furthermore, because the intestinal environment track is rich in a variety of nutrients, the use of a complex medium such as MRS medium rather than a minimal medium is a good way to understand the prebiotic activity of oligosaccharides. For the same reason, Yeast Mold (YM) without glucose was used in this study for the growth of yeast instead of using yeast nitrogen base, the minimal medium generally used for yeast [24].

*W. cibaria* showed the highest viable cell number at 12 h of incubation, following which the viable cell number decreased. This phenomenon was attributed to the production of acid. It has been reported that gentio-oilgosaccharide, which is synthesized by *Leu. mesenteroides* NRRL B-1426, exerts a prebiotic effect on *B. infantis* and *L. acidophilus* [25] and α-(1,6)- and α-(1,3)-linked oligosaccharides with DP 3, which are synthesized by alternansucrase of *Leu. mesenteroides* NRRL B-21297, exhibit a good prebiotic effect [26]. 

Growth of the six probiotic strains, *Lactobacillus casei*, *L. pentosus*, *L. plantarum*, *Weissella cibaria*, *Bifidobacterim animalis*, and *Saccharomyces cerevisiae*, was better when the CCK-oligosaccharides were used as the sole carbon source compared to fructo-oligosaccharides, which are widely used as prebiotics.

## 4. Materials and Methods 

### 4.1. Bacterial Strains and Culture Conditions

The strain *Leu. lactis* CCK940 (GenBank accession number NZ_NQLF00000000) used in this study was isolated from home-made kimchi [16]. This strain was cultured using Lactobacillus MRS broth (BD, Franklin Lakes, NJ, USA) at 30 °C for 20 h as a seed culture.

### 4.2. Determination of Oligosaccharide Structure

#### 4.2.1. Purification of Oligosaccharides

The oligosaccharides from *Leu. lactis* CCK940 were produced in optimized fermentation conditions and purified as described in a previous report [14].

#### 4.2.2. Thin-Layer Chromatography (TLC)

Samples were spotted onto silica gel 60 F254 (Merck, Darmtadt, Germany), and the gel plates were developed twice using 2:5:1.5 nitromethane (Sigma-Aldrich, St. Louis, MO, USA):*n*-propyl alcohol (Samchun, Gyeonggi, Korea):water. The developed TLC plate was dipped in 0.3% (*w*/*v*) *N*-(1-naphtyl) ethylenediamine dihydrochloride (Sigma-Aldrich) and 5% (*v*/*v*) sulfuric acid (Duksan, Seoul, Korea) in methanol (CARLO ERBA Reagents S.A.A., Val de Reuil, France), and then baked at 121 °C for 5 min. Glucose polymers (G1–G7, of which the DPs are 1–7, respectively) purchased from Carbosynth Co. (Berkshire, UK) were used as standard sugars.

#### 4.2.3. HPAEC-PAD Analysis

The CCK-oligosaccharides produced by *Leu. lactis* CCK940 were analyzed using a HPAEC-PAD (DX 500 Chromatography System, Dionex, Sunnyvale, CA, USA) [14]). The *Leu. lactis* CCK940 culture was centrifuged at 27,237× *g* for 1 min and the supernatant was analyzed by HPAEC-PAD (Dionex) with a CarboPac PA-1 column (4 × 250 mm, Dionex) and a CarboPac PA-1 guard column (4 × 50 mm, Dionex). The flow rate was 1.0 mL/min, and the mobile phase used for oligosaccharide separation was 150 mM sodium hydroxide for the first 20 min; subsequently, 600 mM sodium acetate (in 150 mM sodium hydroxide) was applied with a gradient of 60:40 to 0:100 from 20 to 25 min, and 150 mM sodium hydroxide was used from 25 to 40 min (sodium hydroxide, Fisher Scientific, Hampton, NH, USA; sodium acetate, Sigma-Aldrich). Ten microliters of each sample was injected for each analysis.

#### 4.2.4. Size Exclusion HPLC Analysis

The size of the CCK-oligosaccharides produced by *Leu. lactis* CCK940 was analyzed using size exclusion chromatography. The CCK-oligosaccharides (0.1%, *w*/*v*) were dissolved in distilled water and the soluble CCK-oligosaccharides were analyzed by HPLC (UltiMate^TM^ 3000 RSLC nano system, Thermo Fisher scientific, Inc., Waltham, MA, USA) equipped with an OHpak SB-802.5 column (8.0 × 300 mm, Shodex, New York, NY, USA). The flow rate was 0.4 mL/min, column oven temperature was 35 °C, the mobile phase used for CCK-oligosaccharides separation was filtered and distilled water, and they were detected by RI (RI-101, Shodex). One hundred microliters of sample was injected for analysis. Glucose polymers (DP 1–7) purchased from Carbosynth Co. (Berkshire, UK) were used as a standard sugar.

#### 4.2.5. Analysis of Sugar Composition

The CCK-oligosaccharides were hydrolyzed with 4 M TFA at 121 °C for 2 h. TFA was then removed by evaporation under gentle N_2_ gas flow. The sugar components of the CCK-oligosaccharides were analyzed by HPAEC-PAD and TLC.

#### 4.2.6. Composition of Glycosidic Bonds by Enzymatic Analysis

CCK-oligosaccharides were treated with various carbohydrate-hydrolyzing enzymes to determine the composition of the glycosidic bonds. CCK-oligosaccharides (1%, *w*/*v*) were treated with the following enzymes and concentrations: 10 mU of α-amylase, 100 mU of α-glucosidase, 520 mU of amyloglucosidase (Sigma-Aldrich), 1.4 U of pullulanase M1, 10 mU of lichenase (Megazyme, Chicago, IL, USA), 100 mU of β-glucosidase, and 10 mU of β-1,3-d-glucanase (Sigma-Aldrich). The CCK-oligosaccharides were reacted with enzymes at 37 °C for 1 h and the products were determined by TLC.

#### 4.2.7. Analysis of Linkage Ratio by Proton Nuclear Magnetic Resonance (^1^H-NMR) Spectroscopy

The relative abundance of α-1,4 and α-1,6 linkages in the CCK-oligosaccharides was determined by 400 MHz ^1^H-NMR spectroscopy (JeolJNM-LA400 with LFG, JEOL, Tokyo, Japan) and Delta NMR Processing and Control Software version 5.3 (JEOL USA, Inc., Peabody, MA, USA). Freeze-dried samples (10 mg/mL) were first dissolved in deuterium oxide (D_2_O) and then freeze-dried again. Samples dissolved in D_2_O (10 mg/mL) were analyzed by ^1^H-NMR. ^1^H-NMR spectra were collected at 80 °C.

### 4.3. Prebiotic Effects

#### 4.3.1. Selection of Probiotic Strains

A total of 24 probiotic strains were obtained from the Korean Culture Center of Microorganisms (KCCM, Seoul, Korea); *B. adolescentis* KCCM 11206, *B. animalis* KCCM 11209, *B. bifidum* KCCM 12096, *B. breve* KCCM 42255, *B. longum* KCCM 11953, *Enterococcus faecium* KCCM 12118, *L. acidophilus* KCCM 32820, *L. casei* KCCM 12452, *L. fermentum* KCCM 35469, *L. helveticus* KCCM 40989, *L. johnsonii* KCCM 41274, *L. paracasei* KCCM 40995, *L. pentosus* KCCM 40997, *L. plantarum* KCCM 12116, *L. rhamnosus* KCCM 32405, *L. salivarius* KCCM 40210, *Lactococcus lactis* KCCM 40104, *Leuconostoc citreum* KCCM 12030, *Leu. mesenteroides* KCCM 11325, *Pediococcus pentosaceus* KCCM 11902, *Streptococcus thermophilus* KCCM 35496, and *W. cibaria* KCCM 41287. These strains were cultured in MRS medium in the presence or absence of prebiotics at 37 °C for 48 h. *S. cerevisiae* KCCM 50549 and *Zygosaccharomyces rouxii* KCCM 12066 were cultured using YM medium at 30 °C.

#### 4.3.2. Growth Conditions of Probiotic Strains

To determine the prebiotic effects of the CCK-oligosaccharides produced by *Leu. lactis* CCK940, selected probiotic strains were incubated with medium containing CCK-oligosaccharides as the sole carbon source. m-MRS and modified YM (m-YM) had no carbon sources (Table 2 and Table 3). For lactic acid bacteria, m-MRS was used as a negative control, commercial Lactobacillus MRS (MRS containing 2% (*w*/*v*) dextrose) was used as a positive control, MRS with 1% (*w*/*v*) FOS, MRS with 1% (*w*/*v*) CCK-oligosaccharides (CCK), m-MRS with 1% (*w*/*v*) FOS, and m-MRS with 1% (*w*/*v*) CCK media were used. For yeast strains, m-YM was used as a negative control, commercial YM (YM containing 1% (*w*/*v*) dextrose) was used as a positive control, and m-YM with 1% (*w*/*v*) FOS, and m-YM with 1% (*w*/*v*) CCK media were used.

#### 4.3.3. Microbiological Analysis

A colony of lactic acid bacteria was seeded in MRS broth and incubated at 37 °C for 24 h. Additionally, one colony of yeast was seeded in YM broth and incubated at 30 °C for 24 h at 250 rpm. One percent (*v*/*v*) culture broth was inoculated in various media. *Bifidobacterium* sp., lactic acid bacteria, and yeast were incubated in anaerobic, facultative anaerobic, and aerobic conditions, respectively. Culture broth was sampled at 0, 12, 24, and 48 h. *Bifidobacterium* sp. and lactic acid bacteria were spread on MRS agar plates and yeast was spread on YM agar plates.

### 4.4. Statistical Analysis

Data are presented as mean ± standard deviation (SD) from triplicate experiments. Statistical analyses were performed using SPSS 23 (SPSS Inc., Chicago, IL, USA). Statistical significance between groups was determined by one-way analysis of variance (ANOVA), followed by Duncan’s multiple range test (*p* < 0.05). 

## 5. Conclusions

To the best of our knowledge, this is the first study to investigate the gluco-oligosaccharides produced from *Leu. lactis* strains, although several studies have been conducted to examine the gluco-oligosaccharides produced by other lactic acid bacteria. The CCK-oligosaccharides found were novel oligosaccharides based on their structure, and we found that the glycosidic linkages were composed of 77.6% α-1,6 and 22.4% α-1,4. The CCK-oligosaccharides significantly promoted the growth of *L. casei*, *L. pentosus*, *L. plantarum*, *W. cibaria*, *B. animalis*, and *S. cerevisiae*, the major probiotic strains used in the food industry. The prebiotic activity of the CCK-oligosaccharides was better than FOS, which is a major commercially available prebiotic, in some probiotic strains. The prebiotic activities of CCK-oligosaccharides and FOS were found to be strain-dependent. This showed the potential suitability of the use of CCK-oligosaccharides as prebiotics and their significance in the rapidly growing market for prebiotics.

## Figures and Tables

**Table 1:** Relative abundance (%) of α-1,4 and α-1,6 linkages in the CCK-oligosaccharides and WCS.

	α-1,4 Linkages (%) ^1^	α-1,6 Linkages (%)	Ratio of α-1,4 to α-1,6
WCS	96.2 ± 0.0	3.8 ± 0.0	25.5 ± 0.3
CCK-oligosaccharides	22.4 ± 1.2	77.6 ± 1.2	0.3 ± 0.0

^1^ Percentage was determined by calculating the area ratio from ^1^H-NMR.

**Table 2:** Components of the modified MRS media.

Ingredient	Amount (g/L)
Proteose peptone No. 3	10.0
Beef extract	10.0
Yeast extract	5.0
Polysorbate 80	1.0
Ammonium citrate	2.0
Sodium acetate	5.0
Magnesium sulfate	0.1
Manganese sulfate	0.05
Dipotassium phosphate	2.0

**Table 3:** Components of the modified YM media.

Ingredient	Amount (g/L)
Yeast extract	3.0
Malt extract	3.0
Peptone	5.0

## References

[1] Coutinho P.M., Deleury E., Davies G.J., Henrissat B. (2003). An evolving hierarchical family classification for glycosyltransferase. J. Mol. Biol..

[2] Lairson L.L., Henrissat B., Davies G.J., Withers S.G. (2008). Glycosyltransferase: Structures, functions, and mechanisms. Ann. Rev. Biochem..

[3] Lee S., Hanh N.T.T., Cho J.Y., Kim J.Y., Moon Y.H., Yeom S.C., Kim G.J., Kim D. (2016). Glucooligosaccharide production by *Leuconostoc mesenteroides* fermentation with efficient pH control, using a calcium hydroxide–sucrose solution. Biotechnol. Bioprocess Eng..

[4] Monsan P.F., Ouarné F., Charalampopoulos D., Rastall R.A. (2009). Chapter 10. Oligosaccharides derived from sucrose. Prebiotics and Probiotics Science and Technology.

[5] Naessens M., Cerdobbel A., Soetaert W., Vandamme E.J. (2005). *Leuconostoc* dextransucrase and dextran: Production, properties and applications. J. Chem. Technol. Biotechnol..

[6] Heincke K., Demuth B., Jördening H.J., Buchholz K. (1999). Kinetics of the dextransucrase acceptor reaction with maltose—experimental results and modeling. Enzyme Microb. Technol..

[7] Leemhuis H., Pijning T., Dobruchowska J.M., van Leeuwen S.S., Kralj S., Dijkstra B.W., Dijkhuizen L. (2013). Glucansucrases: Three–dimensional structures, reactions, mechanism, α–glucan analysis and their implications in biotechnology and food applications. J. Biotechnol..

[8] Monchois V., Willemot R. (1999). –M.; Monsan, P. Glucansucrases: Mechanism of action and structure function relationships. FEMS Microbiol. Rev..

[9] Bindels L.B., Delzenne N.M., Cani P.D., Walter J. (2015). Towards a more comprehensive concept for prebiotics. Nat. Rev. Gastroenterol. Hepatol..

[10] de Vrese M., Schrezenmeir J. (2008). Probiotics, prebiotics, and synbiotics. Adv. Biochem. Eng. Biotechnol..

[11] Roberfroid M. (2007). Prebiotics: The concept revisited. J. Nutr..

[12] Costabile A., Fava F., Röytiö H., Forssten S.D., Olli K., Klievink J., Rowland I.R., Ouwehand A.C., Rastall R.A., Gibson G.R. (2012). Impact of polydextrose on the faecal microbiota: A double–blind, crossover, placebo–controlled feeding study in healthy human subjects. Br. J. Nutr..

[13] Putaala H., Mäkivuokko H., Tiihonen K., Rautonen N. (2011). Simulated colon fiber metabolome regulates genes involved in cell cycle, apoptosis, and energy metabolism in human colon cancer cells. Mol. Cell. Biochem..

[14] Lee S., Park G.-G., Jang J.-K., Park Y.-S. (2018). Optimization of oligosaccharide production from *Leuconostoc lactis* using a response surface methodology and the immunostimulating effects of these oligosaccharides on macrophage cells. Molecules.

[15] Chung C.H. (2006). Production of glucooligosaccharides and mannitol from *Leuconostoc mesenteroides* B–742 fermentation and its separation from byproducts. J. Microbiol. Biotechnol..

[16] Finke B., Stahl B., Pfenninger A., Karas M., Daniel H., Sawatzki G. (1999). Analysis of high-molecular-weight oligosaccharides from human milk by liquid chromatography and MALDI-MS. Anal. Chem..

[17] Koepsell H.J., Tsuchiya H.M., Hellman N.N., Kazenko A., Hoffman C.A., Sharpe E.S., Jackson R.W. (1953). Enzymatic synthesis of dextran, acceptor specificity and chain initiation. J. Biol. Chem..

[18] Gidley M.J. (1985). Quantification of the structural features of starch polysaccharides by NMR spectroscopy. Carbohydr. Res..

[19] Dobruchowska J.M., Gerwig G.J., Kralj S., Grijpstra P., Leemhuis H., Dijkhuizen L., Kamerling J.P. (2012). Structural characterization of linear isomalto-/malto-oligomer products synthesized by the novel GTFB 4,6-α-glucanostransferase enzyme from *Lactobacillus reuteri* 121. Glycobiology.

[20] Côté G.L., Steinbüchel A. (2002). Alternan. Biopolymers.

[21] Kothari D., Goyal A. (2015). Gentio–oligosaccharides from *Leuconostoc mesenteroides* NRRL B–1426 dextransucrase as prebiotics and as a supplement for functional foods with anti–cancer properties. Food Funct..

[22] Sanz M., Côté G., Gibson G., Rastall R. (2005). Prebiotic properties of alternansucrase maltose–acceptor oligosaccharides. J. Agric. Food Chem..

[23] Hayek S.A., Ibrahim S.A. (2013). Current limitations and challenges with lactic acid bacteria: A review. Food Nutr. Sci..

[24] Abelovska L., Bujdos M., Kubova J., Petrezselyove S., Nosek J., Tomaska L. (2007). Comparison of element levels in minimal and complex yeast media. Can. J. Microbiol..

[25] Lee S., Park Y.-S. (2017). Oligosaccharide production by *Leuconostoc lactis* CCK940 which has glucansucrase activity. Food Eng. Prog..

[26] Astolfi M.L., Protano C., Schiavi E., Marconi E., Capobianco D., Massimi L., Ristorini M., Baldassarre M.E., Laforgia N., Vitali M. (2019). A prophylactic multi-strain probiotic treatment to reduce the absorption of toxic elements: In-vitro study and biomonitoring of breast milk and infant stools. Environ. Int..

